# Soft bioreactor systems: a necessary step toward engineered MSK soft tissue?

**DOI:** 10.3389/frobt.2024.1287446

**Published:** 2024-04-22

**Authors:** Nicole Dvorak, Zekun Liu, Pierre-Alexis Mouthuy

**Affiliations:** Botnar Institute of Musculoskeletal Sciences, Nuffield Department of Orthopaedics, Rheumatology and Musculoskeletal Sciences, University of Oxford, Oxford, United Kingdom

**Keywords:** mechanical stimulation, mechanotransduction, bioreactors, soft systems, soft robotics, soft sensors, tissue engineering, musculosketal tissues

## Abstract

A key objective of tissue engineering (TE) is to produce *in vitro* funcional grafts that can replace damaged tissues or organs in patients. TE uses bioreactors, which are controlled environments, allowing the application of physical and biochemical cues to relevant cells growing in biomaterials. For soft musculoskeletal (MSK) tissues such as tendons, ligaments and cartilage, it is now well established that applied mechanical stresses can be incorporated into those bioreactor systems to support tissue growth and maturation via activation of mechanotransduction pathways. However, mechanical stresses applied in the laboratory are often oversimplified compared to those found physiologically and may be a factor in the slow progression of engineered MSK grafts towards the clinic. In recent years, an increasing number of studies have focused on the application of complex loading conditions, applying stresses of different types and direction on tissue constructs, in order to better mimic the cellular environment experienced *in vivo*. Such studies have highlighted the need to improve upon traditional rigid bioreactors, which are often limited to uniaxial loading, to apply physiologically relevant multiaxial stresses and elucidate their influence on tissue maturation. To address this need, soft bioreactors have emerged. They employ one or more soft components, such as flexible soft chambers that can twist and bend with actuation, soft compliant actuators that can bend with the construct, and soft sensors which record measurements *in situ*. This review examines types of traditional rigid bioreactors and their shortcomings, and highlights recent advances of soft bioreactors in MSK TE. Challenges and future applications of such systems are discussed, drawing attention to the exciting prospect of these platforms and their ability to aid development of functional soft tissue engineered grafts.

## 1 Introduction

Tissue engineering (TE) has the ambitious goal of producing functional tissue grafts *in vitro* to replace, restore or repair tissues at a site of disease or trauma in patients. Such approach uses a combination of cells and biomaterials cultured in bioreactors, which are *in vitro* environments providing appropriate physical and biochemical cues to support growth and maturation. Typical bioreactors often aim to mimic physiological conditions through precise control of basic biological parameters such as temperature, pH, oxygen concentration and nutrient availability. TE bioreactors typically comprise a main chamber that contains sterile culture medium and hosts a tissue construct, a perfusion system to provide nutrient and metabolite exchange with the construct, and sensors to deliver feedback to a control system which maintains ideal operating conditions.

In musculoskeletal (MSK) TE it is well established that tissues require mechanical stimulation in order to grow and mature. For example, tendons deteriorate dramatically when in the absence of tensile stresses: reducing in size and exhibiting lower Young’s moduli, tensile strength and collagen densities (Schraegle, Millard, and King, 1951; Yamamoto et al., 1993). This degradation results from a lack of activation of mechanotransduction pathways which are critical for maintaining the tissue’s structure and functionality. Mechanotransduction, which refers to the ability of cells to respond to mechanical stimulation by releasing biochemical signals (Ingber, 2006), plays a fundamental role in the development and maintenance of MSK tissues (Felsenthal and Zelzer, 2017). Multiple genes are involved in these pathways, such as the PIEZO protein family, a gene encoding an important mechanoresponsive ion channel. Mutations of PIEZO2 have resulted in arthrogryposis (Hall, 1985) or Marden-Walker Syndrome (Rezaei and Saghazadeh, 2016), which are conditions leading to the formation of excessive connective tissue around the joints and joint contracture.

Studies conducted on primary tenocytes (TC) evidenced two major routes of mechanotransduction; cytoskeleton deformation and cell-matrix interactions (Wang et al., 2018). The cytoskeleton is a dynamic network of interlinking protein filaments present in the cytoplasm of all cells, regulating cell morphology and resistance to mechanical deformation (Hardin, Bertoni, and Kleinsmith, 2015). *Cytoskeleton deformation* occurs through the rearrangement of cytoskeleton components, such as actin, intermediate filaments and microtubules, leading to a plethora of downstream effects. For instance, actin polymerizes and depolymerizes rapidly, leading to translocation of molecules into the nucleus, interacting with the Wnt and TGF-β pathways involved in cell differentiation, cell cycle regulation and migration (Clevers, 2006; M. Y. Wu and Hill, 2009). Significant stresses may even affect the stability of chromatin structures inside the nucleus, leading to DNA conformational changes and transcriptional alterations (Tajik et al., 2016). More mechanotransduction pathways are currently being uncovered, such as the role of Phosphoinositide 3-kinase (PI3K) in mechanosensing in the plasma membrane in osteocytes (Danciu et al., 2003), myocytes (Patel et al., 2018) and cancer cells in a response to membrane tension on the actin cytoskeleton and the coupling of tension with intermediate filaments and microtubules (Di-Luoffo et al., 2021). Around the cells lies the extracellular matrix (ECM), an environment which cells interact with, under constant remodelling to control tissue homeostasis. A dysregulated composition, structure and stiffness of ECM often result in pathological conditions, such as fibrosis in MSK tissues (Bonnans, Chou, and Werb, 2014). *Cell-matrix interactions* are enabled by focal adhesions, linking cells’ intracellular actin bundles to the ECM via integrin-containing multi-protein structures (Geiger and Zamir, 2001). Integrins are transmembrane receptors that can be activated upon mechanical load, leading to signal transduction involving pathways such as Erk1/2, TGF beta and Wnt, ultimately regulating factors such as cell adhesion and matrix remodeling (Wang et al., 2018). Beside cytoskeletal deformation and cell-matrix interactions, other mechanisms of mechanotransduction have been proposed, such as those involving gap junctions between neighboring cells. Tenocytes have been shown to remodel gap junctions in response to mechanical stimulation; one study found that while levels of the key protein in gap-junctions, connexin 43, were decreased, connexin 43 mRNA levels were upregulated (Maeda et al., 2012).

**FIGURE 1:**
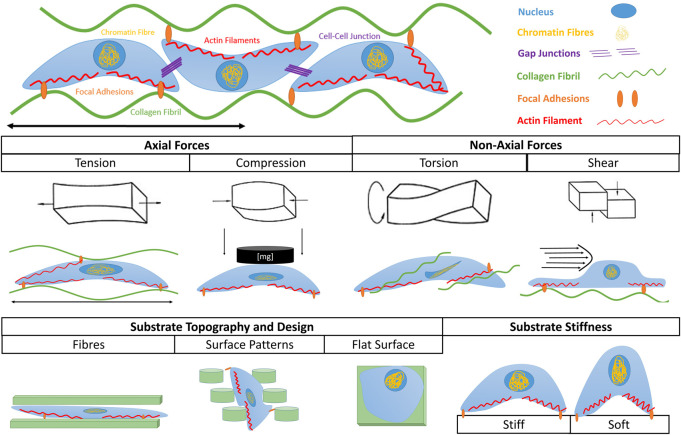
Types of mechanical stimuli leading to mechanotransduction. Cells can sense externally applied axial stresses such as tension and compression, as well as non-axial stresses such as torsion and shear stress. They can also sense internal mechanical cues exerted by substrate topography and substrate stiffness. In each scenario the shape and fate of cells are influenced by the given stimulus.

The importance of mechanical signaling in regulating cell fate, as well as developmental processes *in vivo* has been extensively reviewed by De Belly et al. (De Belly, Paluch, and Chalut, 2022). Despite our existing knowledge, there is a growing need for new approaches and tools (e.g., instruments with high spatiotemporal resolution) able to assess and understand the interplay between mechanical stimulation and cell fate (Wang et al., 2018; De Belly, Paluch, and Chalut, 2022). This is partly because of the lack of existing *in vitro* bioreactor platforms that can enable an in-depth study of the physiological stresses experienced by cells in native tissues. Such stresses include a variety of mechanical stimuli which have been summarized in Figure 1. Passive mechanical stimulation includes cues such as topography and stiffness variations. These have been shown to modulate migration, gene expression and cell fate. (Discher, Janmey, and Wang, 2005; Sharma and Snedeker, 2010; Chao, Hsu, and Tseng, 2014).

Active mechanical stimulation includes tensile, torsional, compressive and shear resulting in stresses. *In vivo,* these types of load exist together in complex loading arrangements which contribute to proper tissue development, and are challenging to model. A detailed description of the different types of mechanical stimulation experienced by MSK cells and tissues is outside of the scope of this article. Further details can be found in existing reviews such as by Sinha et al. (Sinha et al., 2017).

Traditional bioreactors do not currently replicate the variety of physiological stresses. In most cases, they apply basic uniaxial cyclic stresses, either through tension or compression (Kluge et al., 2011; Qin et al., 2015). Combinations of stimuli, such as uniaxial and shear stress (Maeda et al., 2013) or tension and torsion (Altman et al., 2002; Sawaguchi et al., 2010), have also been explored but these studies remain limited despite showing improved biological and biochemical outcomes over single-type stimuli (Altman et al., 2002; Sawaguchi et al., 2010; Maeda et al., 2013). A major obstacle in precisely replicating complex loading conditions with traditional bioreactors is their rigid design. This excludes the possibility of applying relevant multiaxial motion and to take into account the anatomy and size of the tissue of interest. Indeed, tissues vary in size and makeup depending on their anatomical location and attaching muscle: muscles responsible for high power and endurance, such as the *quadriceps femoris* and *triceps surae*, have short and robust tendons, while muscles of precise and delicate nature, such as the finger flexors, have elongated and thin tendons (Bordoni and Varacallo, 2019).

While traditional bioreactors are still widely used, a new class of bioreactor involving soft components is emerging. These aim to address the challenge of multiaxial stresses through the inclusion of soft chambers, soft actuators, soft sensors and their combination. In this review, we examine traditional bioreactor systems and their shortcomings. We also highlight the emergence of soft bioreactors and discuss their potential to apply physiologically relevant multi-axial stimulation, which could ultimately lead to the development of fully functional soft tissues.

## 2 Traditional bioreactors

An overview of traditional TE bioreactors’ components, their role, design characteristics and their limitations is shown in Table 1.

**TABLE 1:** Summarized characteristics for traditional tissue engineering bioreactors.

Bioreactor component	Role	Design Characteristics	Main Limitations/Challenges
Chamber	Host and hold cell-material construct in place	Clamps	Constructs tend to slip. Uneven force transmission
	Deliver nutrients by holding media or providing perfusion through inlets/outlets	Rigid	Poor versatility
			Expensive larger volumes of media
			Poor nutrient distribution through no- or low-flow pockets
			Locally higher shear stress
	Maintain sterility	Linked to actuator	Challenging to fit under microscope
Actuator	Provide programmable, controlled, repetitive uniaxial mechanical stimulation	Stepper motors	Bulky system makes upscaling more difficult and expensive
			Must stay protected from humidity
			Not always incubator compatible
		Uniaxial Stimulation	Not physiologically relevant
		Linked to chamber	Challenging upscale
			Poor versatility
Sensor	Monitor forces and cell culture conditions	External load-cells linked to actuation system	No information on load distribution within the construct
		Stiff and invasive	limited ability for multiaxial stimulation

### 2.1 Chamber designs used in traditional bioreactors

**FIGURE 2:**
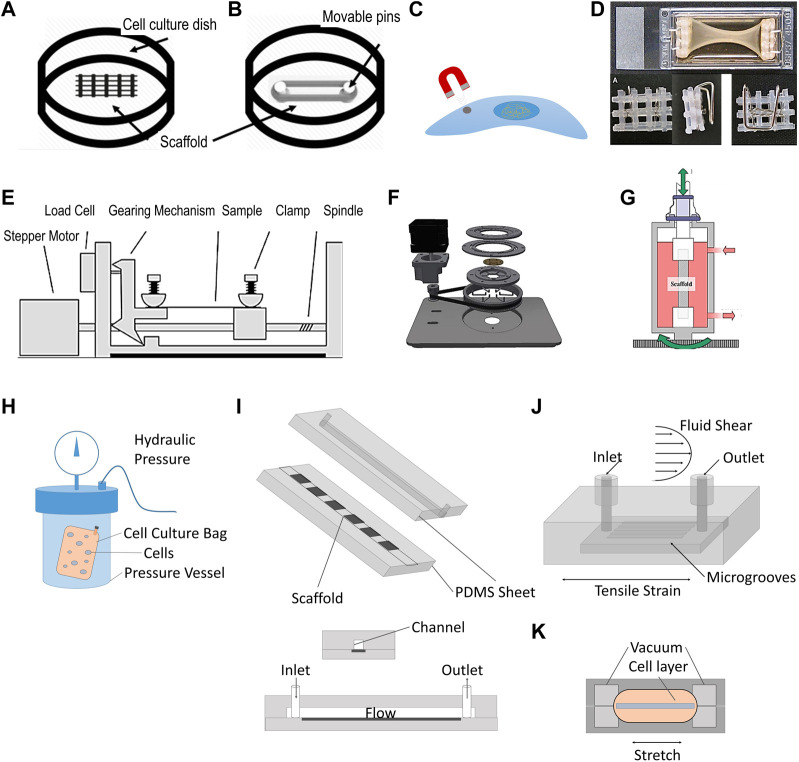
Illustration of traditional tissue engineering bioreactors. **(A–D)** Static stimulation: **(A)** Cell culture on scaffold with topographic cues. **(B)** Cell culture on a scaffold stretched between Teflon pins (Rinoldi et al., 2019). **(C)** Magnetic tweezer technology used to apply a membrane pinch to individual cells (Friedrich et al., 2017). **(D)** Static stretch through flotation bars (Smith et al., 2012). **(E–G)**. Dynamic stimulation: **(E)** Conventional setup of a stepper motor applying repetitive stretch to a clamped sample (figure adapted from Willenberg et al., 2016). **(F)** Example where stepper motor is used to produce radial displacement (Schürmann et al., 2016). **(G)** Torsion bioreactor applied to tendon tissue engineering (Laurent et al., 2014). **(H–K)**. Shear stress and stress combinations: **(H)** Hydrostatic pressure vessel, containing a cell-culture bag with human mysenchymal stem cells (Freeman et al., 2017) **(I)** Example of shear stress bioreactors using medium perfusion (adapted from Kang et al., 2013). **(J)** Bioreactor chamber capable of stretch and shear stress application (Maeda et al., 2013). **(K)** Organ-on-a-chip platform to study effect of mechanical stimulation and shear stress (Huh et al., 2010). Permissions granted where necessary.

Chambers refers to the outermost shell, hosting the tissue construct, culture media, actuators and sensors in a TE experiment. Traditional chambers are made of rigid materials. Many of these include modified Petri-dishes (Raimondi et al., 2018; Rinoldi et al., 2019), single well chamber slides (Smith et al., 2012), and multi-well plates (Reinwald et al., 2015; Feng et al., 2016). Others consist of bespoke rigid boxes with an inlet and outlet for perfusion of culture medium (Laurent et al., 2014). Chamber sizes vary depending on the application, culture duration and perfusion requirements ranging from 0,5 mL for mechanotransduction studies (Kang et al., 2013; Feng et al., 2016) to approximately 40L cylinders for proper tissue engineering studies (Figure 2C) (Peter A. Galie and Stegemann, 2011; Stoffel et al., 2017). Mass and heat transfer, fluid flow, advection and diffusion of nutrients, gas mixing, and reaction rates are important variables to consider when designing a bioreactor chamber and determining its operational variables. Box-shaped non-deformable chambers offer the advantage of easier *in silico* modeling, which can allow for more straight-forward optimization of parameters such as size and perfusion rate (Shakeel et al., 2013; Chapman et al., 2014; Pearson et al., 2015).

Other practical considerations include the type of materials (including adhesives) used in bioreactor chambers, which primarily need to be biocompatible and non-cytotoxic. Materials must also be able to undergo sterilization such as through autoclaving. Common materials for chamber fabrication include acrylic or borosilicate glass, stainless steel and medical grade plastics such as polyethylene or polyvinyl chloride (PVC). Permeability of the material to gases can be a consideration to enable oxygen diffusion into the media, but chambers are more typically equipped with air vents and filters to enable gas-exchange.

### 2.2 Mechanical stimuli applied in traditional bioreactors

#### 2.2.1 Static loading

Static loads are amongst the simplest to apply experimentally, and can be generated in different ways. One approach is to fix a sample under constant tension by wrapping the construct between two adjustable pins (Figure 2B) (Rinoldi et al., 2019). Another strategy is to apply a membrane pinch to individual cells through the application of magnetic tweezer technology (Friedrich et al., 2017) (Figure 2C). A similar approach was used with glass pipettes using suction and a micro-actuator to pull cells from both ends to elongate them (Friedrich et al., 2017). Static tension can also be exerted by clamping a tissue construct (Figure 2D). Static tension has been shown to have a significant impact on cell fate and matrix deposition. For instance, periodontal ligament cells (PDLCs) were exposed to 3% static mechanical strain in a Flexcell tension technology bioreactor for 3 days and 7 days which promoted PDLSCs to differentiate into keratocytes as shown by upregulation of genes such as col I, col III, CD34 (Chen et al., 2018). The Flexcell applies equibiaxial static (or dynamic) tensile strain employing a vacuum onto the edges of a substrate across a dome-shaped loading post.

Static compression can also be applied without the need for bespoke equipment. Feng et al. used metal weights on top of a glass cover positioned on a cell layer (Feng et al., 2016). It was shown that PDLCs suppressed cadherin-11 and col I expression, in addition to a more elongated morphology compared to no compression. This suggested that the cadherin-11/b-catenin complex might play an important role in the signal transduction of mechanical stimulation to biological function in PDLCs. Exerting static compression of 0–50% strain onto adult canine chondrocyte seeded scaffolds resulted in a time- and dose-dependent decrease in protein and proteoglycan production (C. R. Lee, Grodzinsky, and Spector, 2003).

#### 2.2.2 Dynamic uniaxial loading

Dynamic uniaxial stimulation aims to mimic the cyclic strain seen *in vivo*. It is applied at similar amplitudes, frequencies, rates and durations. The most common approach for applying dynamic tension in bioreactors is employing a stepper motor for linear actuation (Figure 2E). The commercially available StrexCell, CellScale T6, DynaGen and LigaGen use this strategy. Cyclic tension applied to bone marrow derived stem cells (BMSCs) seeded on decellularized tendon slices led to a significant increase in gene expression of col I, decorin and tenomodulin, compared to statically stimulated samples (Qin et al., 2015). Stepper motors can also be used to provide radial displacement (Figure 2F) (Schürmann et al., 2016), torsion via a gear transmission (Figure 2G) (Scaglione et al., 2010; Laurent et al., 2014) and dynamic compression, as illustrated by the MechanoCulture TX system developed by CellScale. Another way of applying dynamic tension is through pneumatic systems. This was pioneered by AJ Banes in the 1980s, resulting in the commercially available platforms Flexcell and Bioflex (Banes et al., 1985). Through this approach, localization of nuclei and golgi apparatus in cardiomyocytes were reported to have changed compared to static conditions with cell orientation and morphology being differentially influenced by uniaxial vs. biaxial stimulation (Dhein et al., 2014). In another study, the co-culture of BMSCs with tenocytes treated with 10% mechanical strain for 48 h at a frequenzy of 10 cycles/min showed the upregulation of genes such as COL1A1, COL3A1, alkaline phosphatase, osteopontin, tenascin C and tenomodulin (Song et al., 2017).

Dynamic compression has also been explored through different methods, mainly in the context of cartilage and bone TE. For instance, Freeman et al. have used a compression testing machine to apply a cycling pressure of 10 MPa at a frequenzy of 1hz to a cell-culture bag filled with human mysenchymal stem cells (hMSCs) (Figure 2H). The authors showed that, in the precence of chondrogenic priming, the use of compression (hydrostatic pressure) led to faster osteogenesis compared to static conditions (Freeman et al., 2017). Reinwald et al. subjected chick femur-derived skeletal cell-seeded hydrogels to compressed incubator air, creating a gas–liquid interface with a pressurized gas phase on top of a liquid medium phase. The cell-seeded hydrogels were found to be more regular in shape, significantly denser, and exhibited a greater amount of mineralization compared with the non-stimulated gels, indicating that dynamic stimulation is superior to static stimulation in leading to a more bone-like phenotype (Reinwald et al., 2015).

#### 2.2.3 Application of shear stress

Shear stress is a force acting tangentially to a body such as the sliding of collagen fibers in tendon, or fluid flow experienced by tissues such as cartilage. Shear stress bioreactors (Figure 2I) mainly aim to recapitulate these conditions by applying shear stress to cells, and have been extensively reviewed elsewhere (Thangadurai et al., 2023). In a typical setup, the culture medium is perfused through the bioreactor chamber with a peristaltic pump. Rotating wall vessel bioreactors can also provide such stresses by creating laminar flow conditions around tissue constructs. In such platforms, cells cultured at peak stresses of 3.9 dynes/cm^2^ retained their osteoblastic phenotype and showed significant increases in alkaline phosphatase expression and alizarin red staining by day 7, compared with statically cultured controls (Botchwey et al., 2001). The average wall shear stress experienced by constructs is highly dependent on the choice of bioreactor platform and scaffold design. In a comparative experiment, a spinner flask bioreactor, where tissue constructs are immobilized and exposed to fluid flows generated by a stirrer, led to higher wall sheasr stresses than in perfusion platforms (up to 6.5 mPa and 4.1 mPa respectively) (Seddiqi et al., 2023). Such difference was shown to have an impact on pre-osteoblasts MC3T3-E1, which exhibited reduced collagen and calcium deposition throughout polycaprolactone scaffolds after 7 days in spinner flask bioreactors compared to perfusion bioreactors. The effect of fluid flow can also be studied in microfluidic devices, which are miniaturized bioreactor platforms that aim at studying more closely factors influencing cell proliferation and differentiation. They typically enable high-throughput, a highly controlled microenvironment, and cost efficiency (Kou et al., 2011).

#### 2.2.4 Combining types of loading

To increase the physiological relevance of mechanical stresses, combinations of different types of mechanical stimuli have been studied. Bioreactors capable of applying tension and torsion with independent control have also been developed (Figure 2G) (Laurent et al., 2014; Lee et al., 2013). Lee et al.’s showed that tension and torsion led to an increase in the ultimate tensile strength of decellularized porcine tibialis tendons. Chan et al. tested bovine caudral disc explants with a bespoke 2 degrees of freedom (DOF) dynamic torsion-compression bioreactor to study the mechanobiology of intervertebral discs better (Chan et al., 2013). The group found that complex loading induced a stronger degree of cell death and disc degeneration, compared to 1DOF loading, highlighting the importance of complex loading conditions to study disease progression. More advanced whole joint systems have been mainly used for tribology studies (Beckmann et al., 2018; Liu et al., 2015).

A multiaxial knee-joint bioreactor was developed for commercial use by Regemat3D S.L. It mimics *in vivo* conditions of the knee while applying load, tension and displacement, as well as culture parameters such as pH and temperature. The technology combines 3D bioprinting and multiaxial stimulation to mature engineered articular cartilage. Results showed increased activation of cartilage extracelular matrix proteins such as SOX9 transcription factor, col II protein and aggrecan (ACAN) proteoglycan (Campillo et al., 2022).

Galie and Stegemann developd a perfused bioreactor capable of testing the combination of fluid flow and cyclic tension on cardiac flibroblast seeded collagen hydrogels (Peter A. Galie and Stegemann, 2011). They showed that cross flow significantly increased col III levels over control levels, both in the presence and absence of cyclic strain, whereas cyclic strain alone did not significantly affect col III. The decrease in TGF-β1 expression observed in the samples experiencing cyclic strain alone, was prevented under cross flow combined or not with cyclic strain (P. A. Galie et al., 2012). Maeda et al. built a micro-fluidic chamber with similar capabilities (Figure 2J), leading to greater calcium response and significantly increased intracellular Ca2+ concentration compared to the application of one of these stimuli alone (Maeda et al., 2013). Similarly, Huh et al. have deloped a small PDMS lung-on-a-chip platform to study the effect of those stresses on nanoparticle uptake and inflammatory responses by epithelial cells (Huh et al., 2010) (Figure 2K). The work revealed that preconditioning samples with shear stress led to a more pronounced toxic and inflammatory response to silica nanoparticles than tension alone. It also enhanced nanoparticulates uptake, and stimulated their transport into the underlying microvascular channel, as seen in *in vivo* mice models.

Additional combinations of stresses and their effects can be found in a more comprehensive review by Sinha et al. (Sinha et al., 2017).

### 2.3 Sensors used in traditional bioreactors

Sensors and control systems are used to obtain real time information on culture conditions and can feedback into a control system for autonomous regulation. Sensors most commonly used in cell culture measure temperature, gas mix, pH, glucose and metabolites such as lactate or ammonia. Such sensors can be off-line, being away from the culturing system and requiring manual sample taking, such as bench top glucose sensors. Alternatively, in-line sensors do not require sample-taking and are directly integrated into the culture system. These are most typically gas mix or oxygen sensors, or glucose/metabolite sensors and function independently without the need for separate sampling of media (Prill, Jaeger, and Duschl, 2014; Schmid et al., 2018; de Bournonville et al., 2019). Force and strain sensors, such as load cells, strain gauge or similar piezoelectric sensors, are also essential in dynamic bioreactors (Mouthuy et al., 2022; Wu et al., 2017).

Although traditional sensors can be as small as a drawing pin and can have certain degrees of flexibility (e.g., optical and chemical sensors such as oxygen, glucose and lactate), they generally tend to be stiff, relatively cumbersome and invasive.

### 2.4 Challenges and shortcomings of traditional bioreactors

The limitations of traditional bioreactor systems are manifold. First, rigid structures such as the chamber’s wall mean that it is challenging to apply physiologically relevant multi-axial stimulation. This limits our advance in TE since combinations of mechanical stimuli clearly highligted the importance of multiaxial physiological stimulation (see sections above).

Current dynamic bioreactor systems are also often cumbersome and expensive. This can be a limiting factor when designing large scale TE studies. Scaling up can be achieved either by making the chamber larger to actuate multiple samples together or by increasing the number of chambers and actuators to actuate them individually (Kluge et al., 2011). In both cases, this can increase the footprint and cost of the setup significantly.

Furthermore, dynamic bioreactor platforms usually use mechanical grips to hold the tissue construct. Such mounting of samples can be challenging as wet constructs can readily slip from the grips. Adjustable grips have been developed to prevent the sample from slipping during tensioning such as spring-loaded clamps (Stoffel et al., 2017). Distributing the clamp’s pressure homogeneously across the sample is also an important consideration. For example, smooth-faced clamps with a pivoting head have proven to ensure equal stress distribution (Kluge et al., 2011).

Another limitation relates to the traditional, rigid, load-cell sensors, which can only provide an indirect approximation of the force applied to constructs. This does not allow for spaciotemporal mapping of local stresses in soft tissues such as tendon, ligament or cartilage. Studies are occasionally accompanied by finite element analysis to model strains actually experienced by the constructs, but these remain difficult to validate (Subramanian et al., 2017).

An additional challenge with traditional bioreactors is *in situ* data collection and analysis. Microscopic analyses of the constructs, such as through confocal microscopy, are often performed at an end-point readout. Bioreactor systems with an inbuilt microscopy window have been proposed but dimensions fitting microscopes and working distance of the objective lens are often a limiting factor. Lastly, perfusion of media through rigid boxes can lead to no- or low-flow pockets leading to poor nutrient distribution, poor heat transfer and locally higher shear stresses, all negatively affecting cell culture.

## 3 The emergence of soft bioreactors

While TE bioreactors have seen little innovation for decades, recent years have shown the emergence of one particularly interesting and potentially disruptive feature, the softnesss of major bioreactor components such as the chamber, actuators and sensors.

### 3.1 Soft chambers

**FIGURE 3:**
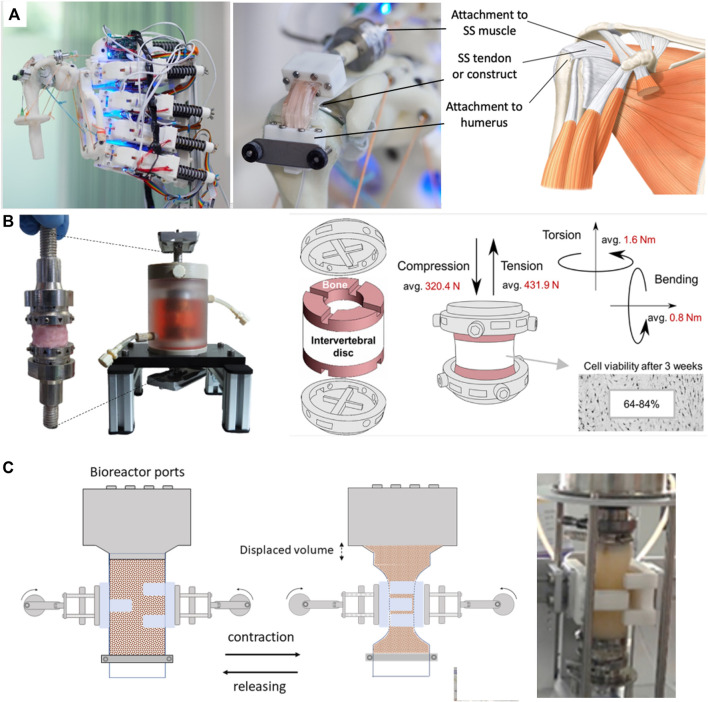
Soft bioreactor chambers. **(A)** Flexible bioreactor chamber proposed for tendon TE positioned on humanoid robotic shoulder, capable of abduction-adduction (Mouthuy et al., 2022). **(B)** Soft intervertebral disc bioreactor using a silicone chamber, offering 6DOF (Secerovic et al., 2022). **(C)** Constriction bioreactor for solid state fermentation using a soft tubular chamber. Though not strictly used for TE, this bioreactor is highlighted for its potential lessons in soft bioreactor design (Hernalsteens, Cong, and Chen, 2022). Permissions granted where necessary.

Soft chambers simply consist of a flexible shell as opposed to the rigid walls seen in traditional bioreactors. Through being flexible, they are able to move with the actuation system and to undergo multiaxial stimulation such as twisting, bending, tensioning or compressing. Mouthuy et al. recently proposed a flexible soft chamber to stimulate tendon constructs on a robotic arm platform (Mouthuy et al., 2022). The chamber, consisting of a thin tube of transparent polyurethane membrane, was remaining leakproof and capable of maintaining sterility under repeated abduction adduction motions. It was shown that the membrane’s contribution to load bearing was negligible, meaning that the applied stresses were mostly directly transmitted to the hosted tissue construct (Figure 3A). Interestingly, the soft chamber was independent from the actuation system., meaning that it was possible to run several chambers in parallel during rest and to attach them to the robotic arm when required. The paper also anticipated that the chambers have the potential to be positioned at different anatomical locations on the robotic arm, as well as on different mechanical platforms such as uniaxial stages.

Using a different approach, Secerovic et al. developed a soft bioreactor chamber aimed at invertebral discs, capable of movement in 6 DOF, strongly resembling the kinematics of the spine (Secerovic et al., 2022) (Figure 3B). Specifically, the platform enabled movements forward/backward, up/down, left/right, as well as changes in orientation through rotation. The chamber consisted of a custom-made polycarbonate structure wrapped by a soft silicon membrane. When compared to a traditional rigid uniaxial bioreactor, invertebral discs were significantly more viable in the multiaxial bioreactor.

Hernalsteens et al. developed a constriction bioreactor for solid state fermentation (Figure 3C). Though not strictly used for mechanical stimulation of soft tissues, this bioreactor is highlighted for its potential lessons in soft bioreactor design. It consists of a silicon tube with flexible walls which enabled mixing via an external actuator, constricting the chamber (Hernalsteens, Cong, and Chen, 2022).

Stoffel et al. developed a knee-joint bioreactor to investigate cell-seeded implants under reproducible, physiological conditions (Stoffel et al., 2017). The knee joint module was mounted into a commercially available axial torsional testing device. To successfully apply human walking cycles, the chamber consisted of a flexible latex sleeve clamped by an elastic band, lodged between acrylic glass and a base plate. The medium was replaced manually while gas exchange was built into the system. The load was measured by an axial-torsional load transducer, a built-in component of the machine.

### 3.2 Soft actuators

**FIGURE 4:**
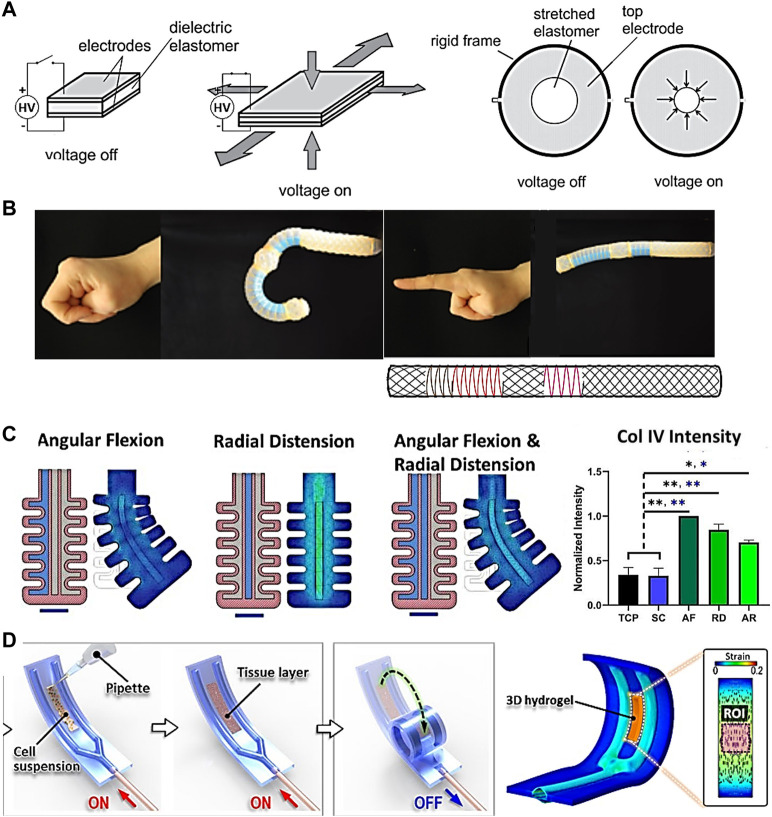
Soft actuators with potential for TE applications. **(A)** Dielectric elastomer actuator capable of constriction mimicking pulsatile contractile motion of the intestinal barrier through rhythmically contracting and relaxing a central cell culture well (Cei et al., 2017). **(B)** Hydraulic soft actuator capable of mimicking the index finger and thumb through bending, extending, expanding and twisting (Connolly, Walsh, and Bertoldi, 2017). **(C)** Artery soft actuator seeded with mesenchymal stem cells, connected to a pneumatic controller, and multiaxially stimulated. Col IV expression was significantly upregulated under mechanical stimulation compared to tissue culture plastic (TCP) and static control (SC) (Images adapted from Fell et al., 2021). **(D)** Soft constrictor actuated pneumatically and capable of dynamic bending motion (Paek et al., 2021). Permissions granted where necessary.

Soft actuators are structures powered by electrical current, hydraulics or pneumatic soft materials that move upon opening or closing valves. Cells can be grown in intimate contact with these materials, allowing more precise transmission of force and strains. Soft actuators have been mostly developed as part of the field of soft robotics, a rapidly growing field with the aim to “permit adaptive, flexible interactions with unpredictable environments” (Kim, Laschi, and Trimmer, 2013). In TE, they can be applied to directly stimulate cell material constructs cultured onto their surface or around them. One example of flexible actuators are dielectric elastomer actuators (Figure 4A) (Poulin et al., 2016; Cei et al., 2017), which are electro-responsive elastomers made from acrylic, silicone, rubber or polyurethane (Brochu and Pei, 2010) that undergo deformation upon electrical stimulation. Such actuators led to the development of a radial artificial muscle device, able to rhythmically contract and relax a central cell culture well through an annular actuator, mimicking movement of the small intestine with circular segmental and longitudinal peristaltic contractions (Cei et al., 2017). These systems are able to apply strain to cells and investigate their mechanoresponse in real time (Akbari and Shea, 2012), and can also function as 3D injury models such as for brain or heart (Y. H. Wu et al., 2022; Imboden et al., 2019). The key advantage of dielectric elastomer actuators is that they can be integrated into the substrate, therefore providing a very compact system. They are also optically transparent, making it easy to incorporate real-time dynamic imaging of cells.

Other flexible actuators with the potential for TE applications are those driven by hydraulics or pneumatics. Figure 4B shows an hydraulic index finger and thumb actuation platform using elastomeric tubes surrounded by an arrangement of fibers, designed with the help of mathematical models, and capable of bending, extending, expanding and twisting (Connolly, Walsh, and Bertoldi, 2017). While cells have not yet been incorporated, it has the potential to stimulate cells in a physiologically relevant way. Furthermore, the algorithm reported may be useful for producing designs for different tissues and overall streamline the production process of soft actuators for different applications.

An example of a pneumatic actuator has been developed by Fell et al., who proposed a bio-hybrid soft robot capable of angular and radial actuation for vascular tissue stimulation (Figure 4C) (Fell et al., 2022). Strains were applied by pumping fluid into a flexible silicone structure. The center of the structure was seeded with MSCs which were subsequently conditioned for 24 h in angular flexion, radial distension, and combined actuation modes. Each regimen induced a unique cytoskeletal orientation as opposed to statically cultured MSCs growing in a disordered manner. Results also showed marked increase in Col IV and α-SMA+ production between mechanically conditioned and static control groups, indicating MSCs progress towards a contractile phenotype of smooth muscle cells in the absence of mechano-activation. Another pneumatic actuator was developed by Paek et al. (Paek et al., 2021). The soft robotic constrictor for *in vitro* modelling of dynamic tissue compression (Figure 4D) was made of PDMS and capable of dynamic bending motion, mimicking the constriction of tubular organs such as blood vessels. The system was tested with different cell types such as primary human endothelial cells, fibroblasts, and smooth muscle cells, leading to physiological changes in their morphology due to the applied force. Prior to this work it had been challenging to simulate the contraction of a lumen, suggesting that soft actuators can lead to improved *in vitro* models of complex physiological tissue microenvironments.

It is worth mentioning that most of these studies using soft actuators are currently limited to mechanotransduction work or the stimulation of native tissues, rather than to be used for TE constructs. However, similar approaches could be used to stimulate the maturation of engineered grafts. A good example is the silicone soft robotic sleeve for heart stimulation developed by Roche et al., which actively contracted and twisted to act as a cardiac ventricular assist device (Roche et al., 2017). The pneumatic system was controlled by the native cardiac cycle and adapted the actuation to deliver disease-specific assistance.

### 3.3 Soft sensors

**FIGURE 5:**
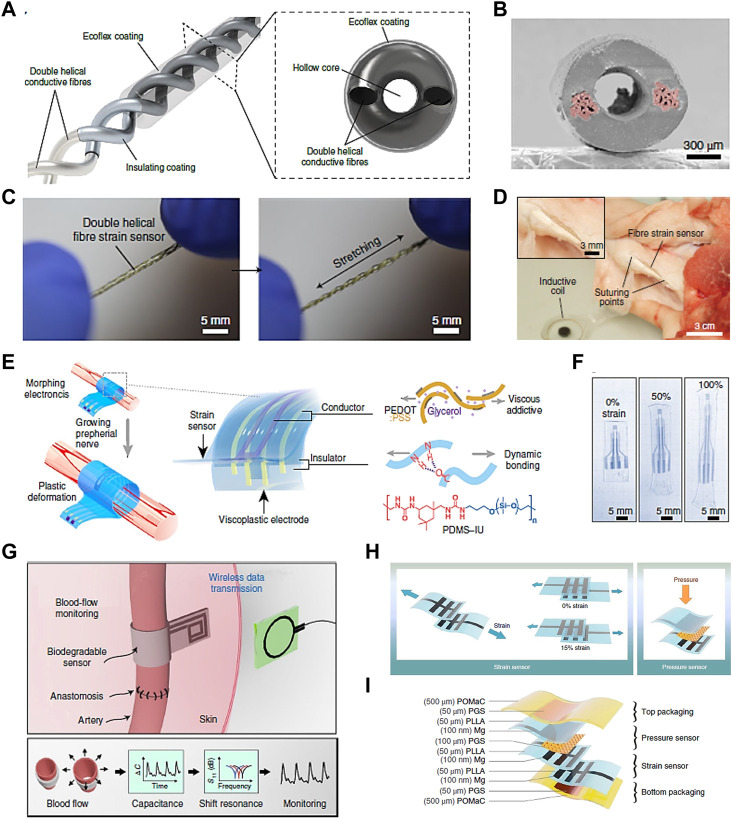
Soft sensors with potentia for TE applications. **(A)** Capacitive strain sensor made with double helical stretchable conductive fibers and corresponding cross-sectional view, **(B)** SEM image of the section morphology of the sensor. **(C)** Photographs of the sensor under initial and stretched conditions, and **(D)** photograph of the fiber sensor sutured onto a pig tendon (Jaehong Lee et al., 2021). **(E)** Schematics of a strain sensor used in neuromodulation and corresponding device components. **(F)** Photographs of the device under the strain deformation of 0%, 50% and 100% (Y. Liu et al., 2020). **(G)** Illustration of a biodegradable, flexible and passive arterial-pulse sensor and corresponding sensing concept by converting vessel diameter change into electrical capacitance (Boutry et al., 2019). **(H)** Illustration of a sensing device based on capacitance changes with both strain and pressure sensing functions, and **(I)** corresponding layer-by-layer material and assembly details (Boutry et al., 2018). Permissions granted where necessary.

Soft sensors include a wide range of flexible sensors developed for various applications, such as in robotics and bioengineering. Sensors with high compliance overcome the challenges of poor deformation capacity of conventional rigid sensors (Hegde et al., 2023). They are important to detect various types of signals, including mechanical, chemical and biological, and convert them into measurable electrical signals (Z. Liu et al., 2022; Yi et al., 2022). Soft mechanical sensors are particularly useful and promising to detect the mechanical stress and deformation that local cells are undergoing in real time in MSK TE. In a study by Lee et al., a stretchable fiber sensor based on capacitance changes was constructed from intertwined double helical conductive fibers (Figure 5A) (Jaehong Lee et al., 2021). Figure 5B shows that the conductive components as two electrodes are separated by the nonconductive dielectric layer. The sensor with a double helical structure of 3 turns/cm reveals high flexibility and stretchability (Figure 5C). By taking advantage of the mechanical and biocompatible feature, the fiber-like sensor was sutured onto the Achilles tendon of a pig to detect tissue deformations by detecting connective tissue strain (Figure 5D) (Jaehong Lee et al., 2021).

Another implantable, biocompatible strain sensing device with morphing function was developed for neuromodulation in growing tissue (Figure 5E) (Y. Liu et al., 2020). The device exhibited shape retention elastic behavior at strains up to 100% (Figure 5F). The electronic device with outstanding morphing ability and minimal mechanical constraint allowed chronic electrical stimulation and strain sensing in a rapid-growth rat tissue without function deterioration, and offered a new route for future electronic and regenerative medicine. Besides, a capacitance-based pressure sensor made with biodegradable and soft materials was fabricated for the application of monitoring blood flow (Figure 5G) (Boutry et al., 2019). The sensing function was implemented by converting bloodstream-induced arterial deformation into the sensor’s capacitance changes. It showed potential in the monitoring of tissue regeneration and reconstructive surgery by taking advantage of its sensibility and biodegradability. It overcomes the challenge of non-biodegradability of most reported implantable sensing devices, which might bring about sensing deterioration or the need for a second operation. By implanting the device into the patients, blood flow data could be monitored in real time with efficiency and accuracy. A more meaningful vision is analyzing the blood flow data via deep learning system, thus achieving large digital healthcare of patients.

In addition to a single sensing function, a sensing device with multifunctional sensing ability would be very attractive in MSK TE. Boutry et al. proposed a device with both strain and pressure sensing ability without sensing interference with one another (Figure 5H) (Boutry et al., 2018). It showed excellent biocompatibility without reported cytotoxic effects by culturing with CD68-positive cells for 8 weeks. The strain and pressure sensing functions maintained stable after 3.5 weeks in a rat model, which would be suitable for real-time monitoring of tendon healing (Figure 5I).

Besides stresses and strains, soft sensors able to measure biochemical signals become increasingly attractive (Brunner et al., 2021). Lee et al. proposed a smart bioreactor equipped with fully integrated wireless multivariate sensors for real-time monitoring of stem cell cultures (Jimin Lee et al., 2024). This system enabled continuous monitoring of pH, dissolved oxygen, glucose levels, and temperature in a soft culture chamber through the integration of soft sensors in its walls. Such advancements pave the way for the widespread adoption of soft sensing systems, facilitating large-scale, cost-effective, reproducible, and high-quality engineered cell manufacturing, thus expanding their potential for broad clinical applications.

### 3.4 Combining soft components to address the challenges of traditional bioreactors

**FIGURE 6:**
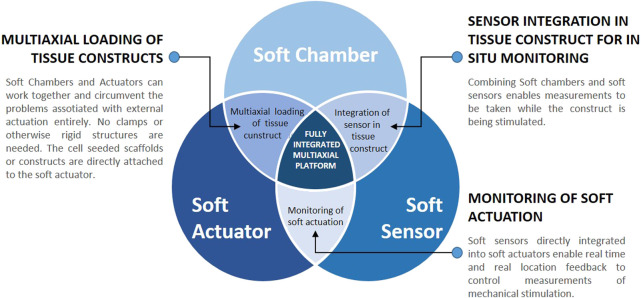
Soft systems as individual entities and their potential synergies. Combining soft chambers and soft actuators could lead to improved multiaxial loading of tissue constructs. Soft chambers could be combined with soft sensors, leading to a fully integrated sensing mechanism, capable of *in situ* real-time measurements of the stresses and strains applied. Combining the soft sensors with soft actuators would greatly improve the understanding and further development of soft actuators. Including all three soft components into one fully integrated multiaxial platform would bear all of these advantages and is likely to lead to the engineering of MSK grafts with improved functionality.

Considering the importance of mechanical stimulation for tissue maturation, the limitations of traditional (rigid) bioreactors and the new advantages of soft components (chambers, actuators and sensors), soft bioreactors are likely to become a promising, if not necessary, step to improve functionality of tissue engineered constructs. This could help MSK engineered grafts to become a realistic therapeutic strategy in clinics (Figure 6). Integrating soft chambers with soft strain sensors would enable real time measurements and improved spatial resolution to uncover subtle local differences in strain distribution. Such combinations are already under development, although not yet in the context of TE (Tric et al., 2017; Jimin Lee et al., 2024). Soft sensors embedded in soft actuators, would provide additional advantages, such as close monitoring of stresses and strains. The combination of soft actuators and soft chambers could sidestep challenges associated with external actuation entirely, such as to make all clamps obsolete. Collectively, the combination of soft chambers, sensors and actuators into a fully integrated soft bioreactor could lead to a system capable of delivering controlled multi-axial stimulation, and of measuring *in-situ* stresses and strains in real time. The cost and scalability of bioreactor platforms would also improve dramatically, as soft components are expected to be more affordable and to have a smaller footprint than their rigid traditional equivalent. Such comprehensive platform is therefore likely to enable the production of engineered tissues with improved functionality. It would also improve our understanding of mechanotransduction through the application of stresses with enhanced physiological relevance, enable the study of different loading regimes and physiotherapy protocols and expand the applicability of bioreactor platforms to different types of tissues (including diseased ones). Soft bioreactor platforms would also greatly contribute to the field of biomechanics, through understanding the importance of mimicking multiaxial movement, anatomical features and tissue biomechanics *in vitro*.

### 3.5 Future technical challenges for soft bioreactors

#### 3.5.1 Materials for soft chambers

There are several important required and desirable properties for flexible bioreactor chambers. These include resistance to cracks, ruptures, decomposition or leaching due to the stresses resulting from the mechanical stimulation. The chamber’s material should also interfere as little as possible with the provision and measurement of stresses. Other desirable properties include the ability to be gas permeable to enable gas exchanges and be optically transparent to enable microscopic observations. Soft polymers such as PDMS (Cei et al., 2017) are therefore promising for this application, although shaping them through molding can be challenging (e.g., obtaining thin films with PDMS). Additional material candidates are commonly used in medicine, such as silicone, polyvinylchloride, polyethylene, polypropylene, polymethylmetacrylate, and polyurethane (Yang et al., 2013; Pandey et al., 2016; Secerovic et al., 2022). These materials can be shaped and sealed into flexible membranes or bags through melting, ultrasonic welding, UV/LED curing systems or medical grade glues such as epoxies.

#### 3.5.2 Assembly of parts and additional ports

Maintaining sterility and proper adhesion while assembling parts and ports is an important challenge in the development of soft bioreactors. For instance, it is possible to position ports on large flexible membranes commonly seen on bioprocess or medical fluid supply bags. However, this is more difficult in smaller soft chamber bioreactors used in TE or mechanotransduction studies. Mouthuy et al. confined a soft chamber with solid 3D printed parts featuring inbuilt inlets/outlets. Medical grade epoxy resin was used as a sealant (Mouthuy et al., 2022).

#### 3.5.3 Attachment of tissue constructs

Traditionally bioreactors make use of grips or clamps to mount tissue constructs. However, these are cumbersome and typically penetrate the bioreactor’s rigid walls, increasing the risk of microbial contamination. Anchoring tissue constructs in soft chambers with mechanical grips is more challenging, given the small space available and the challenge of sealing soft walls. Using other methods such as biocompatible adhesives might be more appropriate. Mouthuy et al. used epoxy resin to firmly anchor the cell carrier into 3D printed inserts, securing the soft chamber. A disadvantage with the use of adhesives is that tissue constructs need to be cut off from the resin bed following culture. Furthermore the resin can penetrate porous scaffolds if its viscosity is too low.

#### 3.5.4 Integrating complex sensors

Soft sensors able to assess the state and conditions that local cells experience in the chamber are highly desireable (Lee et al., 2024). This includes biochemical sensors, such as for measuring concentrations of oxygen, glucose and lactate, as well as mechanical sensors to quantify strains and stresses. Sensor matrices (2D or 3D) might be particularly useful to map properties spatially across the constructs, but integrating such sensors and their electronic components (for data measurement and transmission) without interfering with the tissue and/or the mechanical stimulation remains a challenge. Further developments are necessary to address the current lack of selectivity to specific signals (e.g. differentiation between shear and tension) of these promising sensors (Liu et al. 2021; Liu et al. 2022).

## 4 Other opportunities generated by soft bioreactor systems

### 4.1 Improved studies on biomaterials, mechanotransduction and drugs

Increasingly, evidence shows that *in vitro* multiaxial stimulation leads to different outcomes over uniaxial stimulation (Hornberger et al., 2005; Chan et al., 2013; Fatihhi et al., 2015). This was also shown *in silico* by Fatihhi et al., who used a computational modeling approach to indicate that multiaxial loads reduced the fatigue life of trabecular bone more than five times compared to uniaxial compressive loads (Fatihhi et al., 2015). This suggests that applying multiaxial stimulation to implants and biomaterials to assess their performance might help to accelerate their development and translation to clinical applications. This would be particularly valuable for smart implantable biomedical textites with sensor functions, which are of increasing interest but lack suitable physiological envinronments for their evaluation (Z. Liu and Mouthuy, 2024). Multiaxial stimulation to cell material constructs will also improve our understanding of cellular response to the physiological mechanical stimulation. The implications of better models of mechanotrasduction are plentiful and highly promising, such as to increase our knowledge of disease and repair mechanisms. Studies of joint diseases such as Ehlers Danlos Syndrome, Osteoarthritis and rheumatic diseases could greatly benefit from such models. Similarly, another benefit of soft bioreactors is the improved physiological testing of existing or emerging drugs for treating MSK conditions.

### 4.2 Reduction of animal use

Through their potential of being more physiologically relevant, soft bioreactors could contribute to reducing the number of therapeutic strategies translating too early into animal models. Moreover, by offering greater human anatomical relevance, some of these platforms could reduce the translational gaps in between *in vitro* models, *in vivo* models and clinical applications (Sander et al., 2022).

### 4.3 Versatility of use

In some of the approaches reviewed above, soft chambers were developed to be independent from their actuation system (Mouthuy et al., 2022). These offer several advantages, including easy handling and transportation (e.g., for non invasive monitoring studies), reduced footprint during rest periods, multiple repeats while working with the same actuation system and compatibility with various actuation platforms. (including traditional tensile/compress stages). The application of soft chambers in combination with humanoid robots also opens up the potential of mounting and exercising chambers at different anatomical sites on the same robot.

### 4.4 Improved oxygen supply

A challenge often underestimated when constructing TE bioreactors is the adequate delivery of oxygen to the engineered graft (Place, Domann, and Case, 2017). Materials used in traditional hard-shell bioreactors are rarely permeable to oxygen and therefore rely on a layer of air above the media to enable gas exchange, a media reservoir open to the environment, or additional ports for gas exchange which can cause contamination unless properly filtered. The materials suggested for soft chambers (Sections 3.5.1) are variably permeable to oxygen and therefore passively enable gas exchange without the need for additional ventilation systems, increasing sterility and oxygen exchange. Furthermore, perfusion through flexible chambers can lead to wave-flow, resulting in higher oxygen transfer, leading to more efficient oxygen diffusion than commonly observed through whirlpool flows in shaker flasks (Yang et al., 2013).

## 5 Conclusion

In this review we summarized current shortcomings of traditional bioreactors and discussed the emergence of soft bioreactor systems. For decades traditional mechanical stimulation bioreactors have added great value to MSK TE, but a major limitation is the inability to provide multiaxial stimulation. This is believed to impact the functionaliy of engineered grafts and their subsequent translations to the clinic. Soft bioreactors able to address this shortcoming are progressively emerging and early work has indicated that they are likely to contribute to engineered tissue grafts with improved functionality. We have highlighted 3 components of soft bioreactors such as soft chambers, soft actuators and soft sensors and summarized their advantages and potential problems (Table 2). While already contributing to TE separately, their combination could lead to highly comprehensive platforms that could contribute to the production of soft MSK engineered tissues with improved functionality. Besides leading to the development of better tissue grafts, the opportunities offered by these systems include uncovering mechanotransduction pathways, studying new drug targets, testing different biomaterials and elucidating the disease and repair mechanisms.

**TABLE 2:** Advantages and potential problems of soft features in mechanical stimulation bioreactors.

Soft component	Advantages	Potential problems
Chamber	Flexible walls can undergo multiaxial stresses of various types	Soft materials can more easily crack, rupture, degrade or leach compounds upon stimulation
	Transmission of external loading to engineered graft	Strain/stress transfer can be affected if soft walls are load bearing
		Ability to be leak-proof and assembly can be more challenging
Actuator	Precise strain distribution	Hydraulic/pneumatic designs can be intricate
	Complex motions	Integration with tissue grafts are non-trivial
Sensor	Real time monitoring of mechanical and biochemical signals *in situ*	Current lack of selectivity to specific signals (e.g., no differentiation between shear and tension)
